# Deciphering Variability of PKD1 and PKD2 in an Italian Cohort of 643 Patients with Autosomal Dominant Polycystic Kidney Disease (ADPKD)

**DOI:** 10.1038/srep30850

**Published:** 2016-08-08

**Authors:** Paola Carrera, Silvia Calzavara, Riccardo Magistroni, Johan T. den Dunnen, Francesca Rigo, Stefania Stenirri, Francesca Testa, Piergiorgio Messa, Roberta Cerutti, Francesco Scolari, Claudia Izzi, Alberto Edefonti, Susanna Negrisolo, Elisa Benetti, Maria Teresa Sciarrone Alibrandi, Paolo Manunta, Alessandra Boletta, Maurizio Ferrari

**Affiliations:** 1 IRCCS San Raffaele Scientific Institute, Division of Genetics and Cell Biology, Unit of Genomics for Human Disease Diagnosis, Milan, Italy; 2 Laboratory of Clinical Molecular Biology, Ospedale San Raffaele, Milan, Italy; 3 IRCCS San Raffaele Scientific Institute, Division of Genetics and Cell biology, Molecular Basis of Polycystic Kidney Disease Unit, Milan, Italy; 4 Division of Nephrology and Dialysis A.O. U. Policlinico, University of Modena and Reggio Emilia, Modena, Italy; 5 Depts. Clinical Genetics and Human Genetics, Leiden University Medical Centre, Netherlands; 6 Dept. of Nephrology, Urology and Transplant, IRCCS Cà Granda Policlinico, Milan, Italy; 7 Center for Prenatal Diagnosis and Nephrology, A.O. Spedali Civili, Brescia, Italy; 8 Dept. of Paediatric Nephrology and Dialysis, IRCCS Cà Granda Policlinico, Milan, Italy; 9 Laboratory of Immunopathology and Molecular Biology of the Kidney, Dept. SDB, Padova, Italy; 10 Pediatric Nephrology, Dialysis and Transplant Unit; Department of Women’s and Children’s Health, Padova, Italy; 11 Vita-Salute San Raffaele University, chair of Nephrology, IRCCS Ran Raffaele Scientific Institute, Genomics of Renal Disease and Hypertension Unit, Milan, Italy; 12 Vita-Salute San Raffaele University, chair of Clinical Pathology, Milan, Italy

## Abstract

Autosomal Dominant Polycystic Kidney Disease (ADPKD) is the most common hereditary kidney disease. We analysed *PKD1* and *PKD2*, in a large cohort of 440 unrelated Italian patients with ADPKD and 203 relatives by direct sequencing and MLPA. Molecular and detailed phenotypic data have been collected and submitted to the PKD1/PKD2 LOVD database. This is the first large retrospective study in Italian patients, describing 701 variants, 249 (35.5%) already associated with ADPKD and 452 (64.5%) novel. According to the criteria adopted, the overall detection rate was 80% (352/440). Novel variants with uncertain significance were found in 14% of patients. Among patients with pathogenic variants, in 301 (85.5%) the disease is associated with *PKD1*, 196 (55.7%) truncating, 81 (23%) non truncating, 24 (6.8%) IF indels, and in 51 (14.5%) with *PKD2*. Our results outline the high allelic heterogeneity of variants, complicated by the presence of variants of uncertain significance as well as of multiple variants in the same subject. Classification of novel variants may be particularly cumbersome having an important impact on the genetic counselling. Our study confirms the importance to improve the assessment of variant pathogenicity for ADPKD; to this point databasing of both clinical and molecular data is crucial.

Autosomal Dominant Polycystic Kidney Disease (ADPKD) is the most common genetic nephro-pathology in humans, with a prevalence of 1/400–1000 subjects at birth^1^, and characterized by bilateral renal cyst formation. Cysts develop by massive enlargement of every segment of the nephron resulting in renal failure leading to dialysis or transplant^1^. The observed phenotypic variability in ADPKD involves differences in the rate of loss of glomerular filtration, the age of reaching end-stage renal disease (ESRD), the occurrence of hypertension, symptomatic extra-renal cysts, and subarachnoid hemorrhage from intracranial ‘berry’ aneurysm.

The majority of ADPKD patients carry a germ-line variant in the *PKD1* gene (ADPKD1- MIM173900) on chromosome 16p13, and 15 to 20% of patients harbor a *PKD2* variant on chromosome 4q21 (ADPKD2-MIM613095). Disease manifestations are characterized by a high inter and intra familial variability, raising the possibility of the existence of modifiers for the disease^1^. Clinical symptoms usually do not arise until middle age. Recently, in a minor subset of patients, in whom ADPKD manifests very early in life and presents as a much more severe disorder^2^ it was demonstrated that early and severe ADPKD is not strictly confined to individuals with a *PKD1* variant as previously thought but can also occur in other genetic combinations: patients with a *PKD2* variant^3^, coinheritance in trans of an incompletely penetrant *PKD1* allele with an inactivating *PKD1* variant^4^. In addition, among those cases with early-onset ADPKD, are cases with clinical features sometimes indistinguishable from those with a typical presentation of autosomal recessive polycystic kidney disease (ARPKD) and variants in the *PKHD1* gene^5^.

Until recent, the observation that ADPKD1 causes more severe disease with an average age of 54 years at ESRD (versus 74 years for ADPKD2) was the only correlation between genotype and phenotype^1^. In a more recent paper, authors found a correlation between the type of *PKD1* variant and renal survival. The median age at onset of ESRD was 55 years for carriers of a truncating variant and 67 years for carriers of a non-truncating variant^6^.

Taken all together, these studies highlight the potential high complexity of the genetics of ADPKD and warrant the need for studies aimed at characterizing the different variants identified. The high level of allelic heterogeneity, the various types of variants observed in *PKD1* and *PKD2* rise issues with respect to their classification in particular for rare and novel non-truncating variants. Increasing the knowledge on the spectrum of variants and sharing of data would definitely be helpful to improve our capability for variant classification, correlation to clinical features and stratification of patients.

Molecular analysis of ADPKD has proved difficult for a long time, because of genetic and allelic heterogeneity, for the presence of GC-rich regions and more importantly because the 5′ region of *PKD1*, from exons 1 to 33, is duplicated in six highly homologous pseudogenes, all located in chromosome 16p^7^,^8^. To date, the wider variant scanning diagnostic studies of the entire *PKD1* and *PKD2* genes, has been performed by DHPLC and/or sequencing, in combination with other methods for the identification of large rearrangements^8^,^9^. To date, the four larger studies reported the distribution of *PKD1* and *PKD2* variants in 202 (CRISP, USA)^10^, 700 (GENKYST, France)^9^, 220 (TGESP, Canada)^11^ and 1119 patients (HALT-PKD and CRSP, USA)^12^.

Aim of the present work was to realize a comprehensive description of all the genetic variation of *PKD1* and *PKD2* in a large cohort of Italian patients affected by ADPKD: 643 subjects were analysed by a semi-automated Sanger direct sequencing protocol, followed by multiple ligation probe assay (MLPA) analysis. A second aim was to establish a database for ADPKD following existing standards, linked with international resources and suited for the collection of patient-specific variants and key clinical features.

## Results and Discussion

In the present study, a semi-automated direct sequencing method was applied for detection of variants in *PKD1* and *PKD2* genes in a cohort of Italian patients affected by ADPKD. One of the major difficulties of *PKD1* sequencing is the specific selection of the functional gene due to the presence of six highly homologous pseudogenes. In the present study, we applied protocols previously described by Phakdeekitcharoen B. and coauthors^7^ for the specific amplification of *PKD1* by Long-Range PCR amplification, assuming they are *PKD1*-specific. Nevertheless, to avoid amplification of the *PKD1* pseudogenes, the design of primers, either for the Long-Range or for nested PCR, was verified on a multiple alignment (Lasergene) between PKD1 and the 6 pseudogenes. In order to select primers complementary solely to PKD1, primers were located in regions where a sequence divergence with the pseudogenes is present, with the differences located at the most 3′ nucleotidic position. As an example, we added the Supplementary Figure S1, where the sequencing result of the specific amplification of PKD1 exon 10 with our primers is shown and compared with the result of an unspecific amplification.

By looking at sequencing results, we first considered known variants; we grouped either common or rare variants previously classified as likely neutral as benign variants. A total of 113 *PKD1* and 8 *PKD2* variants were identified, with an average of 11 per patient in *PKD1* and 2 per patient in *PKD2*. In Supplementary Table S1, benign variants observed in *PKD1* are listed with the minor allele frequency (MAF) in our ADPKD cohort, in the 1000 genomes (general and TSI Italian sub-population) and the PKDB classification. By comparing the group of patients and the general populations, none of the variants showed marked differences in their frequencies. We only noticed that sometimes the MAF observed in the group of the patients was more similar to the general MAF reported in 1000 genomes than to that in the TSI subgroup.

In addition to known benign variants, also rare and novel variants were identified; in particular, a total of 701 DNA novel and already described pathogenic variants have been detected: 625 (89.2%) from *PKD1* and 76 (10.8%) from *PKD2*, confirming the marked allelic heterogeneity of these genes. In Table 1, the different classes of variations identified in familial and in sporadic subgroups are listed for each gene. Variants were present in 94.3% of probands (415/440).

Of the total 701 changes, 249 (35.5%) were variants already described (http://pkdb.mayo.edu), 216 (30.8%) in *PKD1* and 33 (4.7%) in *PKD2* (Suppl. Table S2). Among these also variants classified as likely neutral. The remaining 452 (64.5%) were new changes, 409 (58.4%) in *PKD1* and 43 (6.1%) in *PKD2*. Table 2, summarizes the novel variants identified in patients, distributed in classes.

For all the identified variants a classification with respect to pathogenicity was attempted, as described in the methods section. Variants have been classified as definite pathogenic (Supp. Table S3), highly likely pathogenic (Supp. Table S4), likely pathogenic (Suppl. Table S5), of uncertain significance (Suppl. Table S6), or likely non-pathogenic (Suppl. Table S7).

By combining results from known pathogenic variants, current classification and family studies we were able to detect the pathogenic variant in 352 patients.

In Table 3 the types and frequencies of definite and probable pathogenic variants are reported. In *PKD1*, frameshifting variants were the more represented, accounting for 30%, while in *PKD2* the nonsense was the major type with a 35% frequency. By comparing definite pathogenic variants, they were more frequently detected in *PKD2* (76%) than in *PKD1* (66%). In Table 3, data on *PKD1* and *PKD2* from the previous large CRISP^10^. GENKYST^9^. TGESP^11^ and HALT-PKD and CRISP^12^ studies, on 180, 442, 188 and 1034 pathogenic variants, respectively, are reported and compared to the present study. In *PKD1*, truncating variants (grouping frameshift, nonsense, canonic splice site changes and large rearrangements) were the more represented in all the studies, with the TGESP^11^ study displaying the lower percentage (38.3%). On the contrary the GENKYST^12^ study showed the lower frequency of non-truncating variants. In *PKD2* the results were similar with the exception of the TGESP study showing a frequency of variants 2 times higher compared to the other cohorts.

In the present study the overall detection rate was 80% (352/440), with 83% (266/320) in the group of patients with familiarity, and 72% (86/120) in the group of sporadic patients (Table 3).

Among patients with pathogenic variants, 301 (85.5%) carry a *PKD1* variant, and 51 (14.5%) carry a *PKD2* variant, in agreement to previous reports. Moreover, in previous studies, the overall detection rate was higher: 89.1% in the CRISP study^10^. 89.9% in the GENKYST study^9^, 84.5% in the TGESP^11^ study, and 92.4% in the HALT-PKD and CRISP^12^ study. In our cohort, pathogenic variants were not found in 20% (n = 88) of the 440 unrelated patients. Among these, in 25 patients (6%), only known benign variants were present. In the remaining 63 patients (14%), novel variants with uncertain significance were found, mainly missense variants. To explain this lower detection rate, it is possible to argue that the classification system adopted was imperfect, or that our population may be enriched by other classes of pathogenic variants (such as variants located in regulatory regions), not detected. Another explanation may be that in our population the collection of samples was somehow biased. Actually, our cohort was enriched in subjects with an uncertain clinical diagnosis (96/440, 21.8%), since one of the criteria to access genetic testing was the clinical atypical presentation. In this subgroup we obtained a lower detection rate (62/96, 64.6%) of definitely pathogenic variants, a lower proportion of patients with variants with uncertain significance (11, 11.4%) and a higher proportion of patients without variants (23, 24%) who may have other non-ADPKD cystic kidney disease explaining their phenotype.

In order to check the sensitivity and specificity of our genetic testing in relation to the clinical phenotypes, we performed a correlation between the pathogenic variants and the clinical characteristics of patients, when available. Considering the clinical diagnosis and the reason for testing, as reported in Supplementary Table S8, the correlation was done for patients with positive diagnostic criteria in the presence (true positives) or in the absence (false negatives) of a pathogenic variant and for patients not at risk and negative diagnostic criteria in the presence (false positive) or in the absence (true negatives) of a pathogenic variant. We reached 85% sensitivity and 100% specificity.

In order to evaluate the performance of the prediction for missense variants adopted in the present study, a comparison with the results of the prediction reported in PKDB was done. Classification in this study differed from that in the PKDB for 14 out of 83 previously reported missense variants, showing a 83% concordance. As reported in Table 4, among the 14 discrepant variants, 9 were classified as uncertain with our prediction model and likely or highly likely pathogenic in PKDB, indicating a higher grade of uncertainty with our prediction. In 4 cases, variants classified as indeterminate in PKDB resulted as likely pathogenic in this study. Noticeably for three of these our result was only slightly different since a border-line scoring (=14) was obtained. In only 1 case (c.10678G>A) the prediction was completely opposite: likely pathogenic in this study and likely neutral in PKDB; by looking at concurrent variants in the patient it is possible to observe that in this case a truncating variant was present, thus reinforcing probability for the neutral nature of the c.10678G>A variant. Taking into account previous and present classification, a prudent conclusion would be that prediction of missense variants is still imperfect and that a consensus on the criteria for classification of pathogenicity would be useful.

Among the 352 different pathogenic variants in PKD1 and PKD2, 65 (18.5%) of them were found in at least 2 unrelated patients. Table 5 lists the variants that were found in at least 3 unrelated patients, 10 in PKD1 and 4 in PKD2. Of the 14 variants, 8 are single nucleotide substitutions, 5 are small deletions, 1 is a single nucleotide duplication. Only three of them were previously described as frequent/recurrent variants in PKD1: c.2180T>C; c.5014_5015delAG; and c.8311G>A^9^,^10^. The three novel pathogenic variants c.3607C>T and c.11354G>C in *PKD1* and c.637C>T in *PKD2* are very interesting since they may represent Italian clusters but, unfortunately, we were not able to define if the origin of these variants was from a common ancestor.

**Figure 1:**
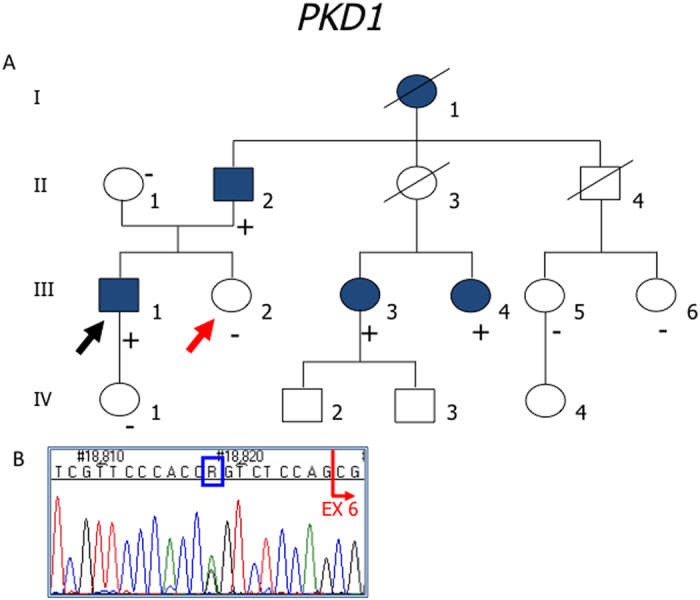
(**A**) In this family, the proband (III-1, black arrow) with ESRD, inherited from the father (II-2) and shared only with affected relatives (III-3, III-4) the intronic *PKD1* variant: c.1202-9G>A. None of the analyzed healthy subjects in the family (II-1, III-2, III-5, III-6, IV-1) had this variant; (**B**) Sequence chromathogram; abolishing of the nearby splice acceptor site was predicted in silico (www.fruitfly.org). Genetic testing was important to support classification of the intronic variant as highly likely pathogenic and to identify the III-2 subject (red arrow) as a potential donor for kidney transplantation in the patient. Unfortunately it was not possible to collect the proper samples for RT-PCR. Filled symbol: ADPKD; empty symbol: asymptomatic; (+) sign indicates a subject heterozygous for the c.1202-9G>A variant; (−) sign indicates a subject without the variant.

**Figure 2:**
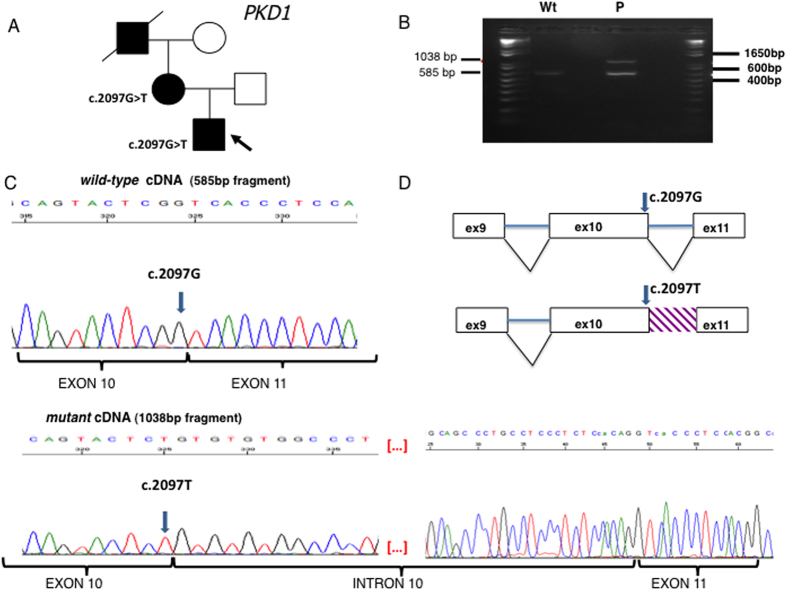
(**A**) Pedigree. In this family, the proband (arrowed) inherited a synonymous *PKD1* variant (c.2097G>T, p. Ser699=) from his mother. (**B**) RT-PCR from whole blood RNA. In the control sample (Wt), only the expected 585 bp fragment was present. In the patient sample (P), an additional 1038 bp abnormal fragment was amplified. (**C**) Sequencing of the regular and abnormal fragments from the patient sample. In the upper panel, the sequence of the 585 bp normal c.2097G allele cDNA showing the adjacent exons 10 and 11, correctly spliced; in the lower panel, the sequence of the abnormal 1038 bp cDNA showing that in the mutated allele c.2097T, intron 10 has been completely retained in the transcript. (**D**) A scheme of the spliced regions in the normal and mutant alleles. Filled symbol: ADPKD; empty symbol: healthy subject.

In some cases, the family study helped us to support the pathogenic role of a variant. This was the case for the c. 1202-9G>A intronic variant (Fig. 1) and for the synonymous c. 2097G>T variant (Fig. 2), in *PKD1*.

**Figure 3:**
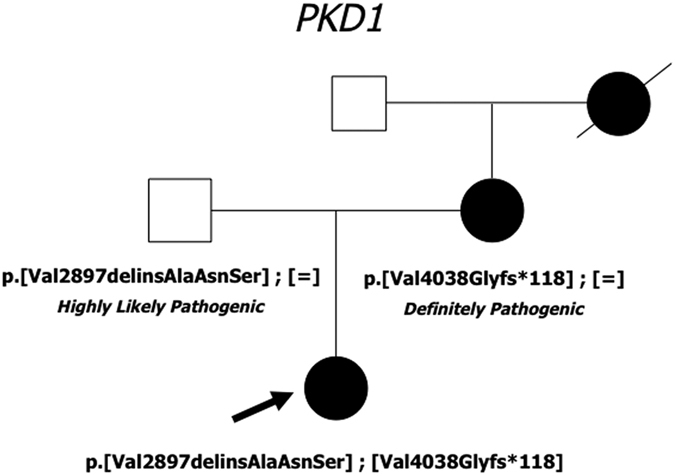
In this family, the proband has inherited from the mother a truncating variant, classified as definitely pathogenic, and from the asymptomatic father a del/ins in-frame variant, classified as highly likely pathogenic with the adopted criteria. The proband developed renal insufficiency much earlier than her mother; a RMI renal examination was advised to the father to evaluate a possible subclinical phenotype. Filled symbol: ADPKD; empty symbol: asymptomatic.

In line with allelic heterogeneity, in many patients more than one candidate variant was observed (Table 6). In the majority of cases, it was not possible to perform segregation analysis within the family, thus the most likely candidate pathogenic variant was inferred based on its nature (truncating, not truncating) and based on predictions, as illustrated in the Methods. Nevertheless, in few cases, more than one variant classified as pathogenic, definitely or likely, novel or described, was present in the same subject, with both of them in *PKD1* or one in *PKD1* and the second in *PKD2* (Table 7). In these cases, various combinations of variants have been found: coexistence of two definitely pathogenic variants or of one definitely pathogenic with a likely pathogenic variant. The simplest interpretation would be to declare as pathogenic only the variant with a definite classification; nevertheless coexistence of a definite variant with another candidate variant has to be taken into account since compound heterozygosity has been already observed in patients with early and severe ADPKD^4^,^5^. Thus, the results of molecular testing for ADPKD have to be considered very carefully and have important implications for genetic counselling since definition of risk in relatives may be cumbersome. In order to define the contribution of each variant to the phenotype, segregation analysis and correlation with the phenotype in the family should be always advised. An example of such a situation is showed in Fig. 3. In this family, segregation analysis showed that the p.Val2897delinsAlaAsnSer in-frame del/ins was inherited from the health father while the definite pathogenic frameshift variant p.Val4038Glyfs*118 was transmitted from the affected mother. The hypothesis that the early onset in the proband may be influenced by the paternal variant, was taken into account and a pathogenic role of the p.Val2897delinsAlaAsnSer, classified as highly likely pathogenic with the criteria adopted^9^, was not excluded. Actually, an argument against its pathogenic role was that it is located near the variant p.2894insAlaAsnSer, previously classified as a polymorphic variant based on segregation studies and on evidence of a recent origin in the human genome^10^. Thus, at the moment, classification of the p.Val2897delinsAlaAsnSer remains doubtful, and further evidences are needed in order to conclude in favour or against pathogenicity.

**Figure 4:**
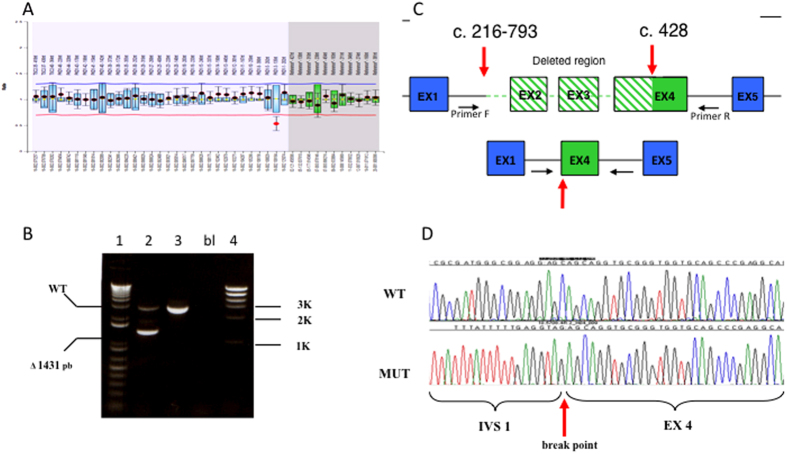
(**A**) MLPA result suggesting the presence of heterozygous deletion of exon 3 in *PKD1*; (**B**) PCR amplification of the deletion borders. In lane 2 the lower fragment is generated by the deleted allele; 2: patient; 3: healthy control; bl: no DNA; 1, 4: molecular weight markers; (**C**) schematic representation of the 5′ region of the PKD1 gene in normal (upper) and deleted (lower) alleles. Primer F-2FLR-5′-ATTTTTTGAGATGGAGCTTCACTCTTGCAGG; primer R-4R 5′-AGCCCTGCCCAGTGTCT. (**D**) Sequence of the deletion boundaries. Direct sequencing revealed the presence of a 1431 bp deletion, extending from nucleotide c.216-793 to c.429 (c.216-793_429del143; http://www.hgvs.org/mutnomen/). The deletion starts 793 bp upstream of exon 2 and stops at position 429 in exon 4, thus causing a frameshift with the production of a truncated putative protein (p.Leu72fs).

Search for large rearrangements by MLPA revealed one case with deletion of the whole *PKD2*, the fifth reported to date^13^,^14^,^9^, two cases with deletion of the whole *PKD1* and one case with a partial *PKD1* deletion (c.216-793_427del1431), described in Fig. 4. This finding is in line with previously reported frequencies (1–3%)^9^,^15^. Nevertheless, we have to notice that commercial MLPA assays, like that used in the present study, do not include probes specific for all exonic regions, therefore we cannot exclude the occurrence of false negative results.

**Figure 5:**
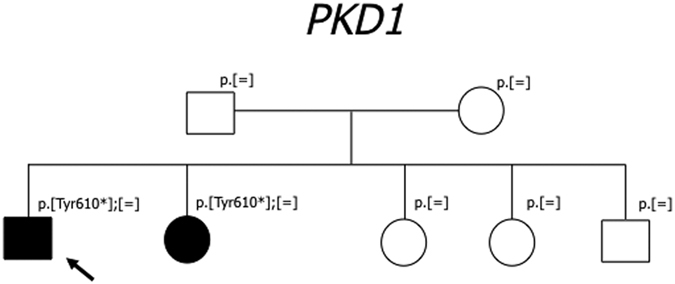
In this family, the proband and his sister are the only ADPKD affected subjects; the proband has kidney and liver multiple cysts, his sister had kidney and liver transplantation at 44 years of age, the parents are healthy and negative to US abdominal examination. Genetic analysis in the family revealed that they did not inherited the truncating variant p.Tyr610*, suggesting the presence of germinal mosaicism in one of the parents. Paternity was confirmed. Filled symbol: ADPKD; empty symbol: healthy.

In Fig. 5 a family with evidence of germinal mosaicism is shown. Somatic mosaicism has been described in 40% of PKD1/TSC contiguous gene deletion syndrome patients, in association with large rearrangements involving the adjacent *PKD1* and *TSC* genes^14^. In our family, the disease was associated with a truncating variant, the presence of two affected siblings suggested a mosaicism in the parents, likely confined to the germ-line and excluded the hypothesis of a *de novo* mutation, with an impact on their reproductive risk.

**Figure 6:**
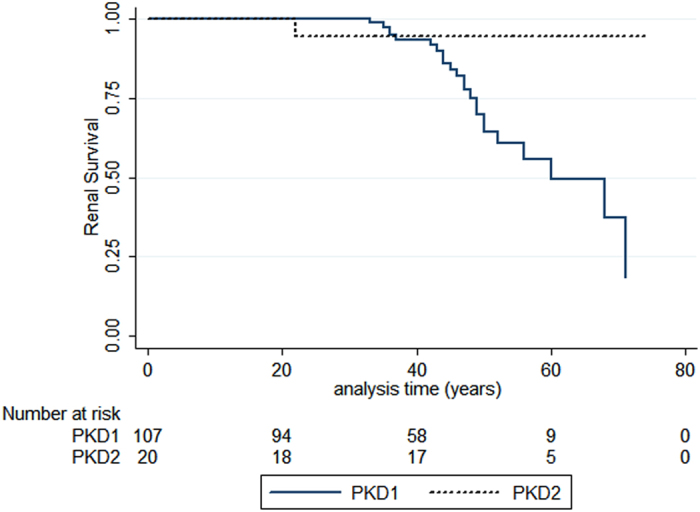
Allelic influence on renal survival. A significant difference in renal survival between patients harboring a *PKD1* pathogenic variant and patients with a *PKD2* pathogenic variant was observed. Log rank test: Chi-square 5.47, p = 0.0194.

Considering the high allelic variability observed in patients, and taking into account the reported correlation between the various classes of pathogenic variants and the onset of ESRD^6^,^11^,^12^, we calculated the proportion of patients with or without ESRD in the subgroups with PKD1 truncating, not truncating, IF indel and PKD2. As shown in Table 8, the proportion of *PKD1* truncating variants is higher in the group of patients with ESRD. By comparing the age at onset of ESRD in patients harbouring *PKD1* or *PKD2* pathogenic variants, we observed a significant difference between the subgroups (*p* = 0.0194 log rank) (Fig. 6), with an earlier ESRD onset in *PKD1* patients. These results are in line with previous observations; nevertheless, in consideration of the retrospective nature of these clinical data, we feel that a more detailed and complete collection of clinical data will be necessary to assess the prognostic value in term of renal survival.

## Conclusions

This is the first Italian study performed on a large collection of 440 probands with ADPKD, aimed at identifying the molecular variability in *PKD1* and *PKD2*. The resulting genetic variants as well as key clinical data have been collected in a database established at the Leiden Open Variation Database^16^. These set data will be relevant for classification of variants as well as for a better description of phenotypes. Furthermore, since the LOVD databases have a webservice (api) the data can be accessed electronically and used to automatically annotate exome/whole genome sequencing data using tools like EBI’s Variant Effect Predictor (WEP).

In this study we added further knowledge and new observations to ADPKD disease by explaining the molecular defect in 352 patients, with the majority of them (208/352) carrying a new pathogenic variant. The large majority of pathogenic variant was constituted by single base substitutions or small in-dels, while large rearrangements displayed a low frequency (<2%). Molecular analysis helped us to confirm the diagnosis in clinically uncertain/atypical cases, to exclude the presence of a variant in donors for kidney transplantation and to offer genetic counselling in at risk families. To this respect, by using genetic testing in relatives of probands with a pathogenic variant, we were able to prove 97 individuals to be free of the disease and 91 individuals diagnosed with ADPKD.

Genetic analysis revealed an extraordinary high degree of allelic variability, especially in *PKD1*; we were able to identify a definite pathogenic variant in 54% of cases. In order to improve counselling to patients and their relatives, we undertook the analysis of the remaining unclassified variants with predictive tools, according to the criteria adopted by Audrezet MP and co-authors^9^. These tools evaluate the effect of variants in light of conservation in a multi-sequence alignment. Data on segregation analysis, co-occurrence with a pathogenic variant, frequency analysis in controls, analysis of transcripts and in-silico evaluation of intronic variants were taken into account to add evidence and were very helpful for counselling. Classification of highly likely or likely pathogenic variants in 26% of patients raised the total detection level to 80%.

In addition to classified variants, other novel variants have been found, but their classification was uncertain with the criteria adopted, especially for missense changes with a score below the fixed threshold (122 in *PKD1* and 7 in *PKD2*). Thanks to the contextual analysis of *PKD1* and *PKD2*, a number of variants in the group with uncertain significance (Suppl. Table S6), when they were the only one present, might be considered as a strong candidate pathogenic variant, nevertheless we did not consider this observation sufficient to assess pathogenicity. In general, classification of missense variants remains cumbersome because it is not feasible to perform a functional study for each variant, sometimes due to experimental limitations, like for Polycystin-1, but more often due to practical and economic barriers. *In silico* predictors of pathogenicity have been developed and have become popular but they often lack standardization and clinical validation. In the present study we applied classification criteria previously described for ADPKD^9^ and compared the results to that obtained by Rossetti *et al*.^10^ for the same missense variants, obtaining a concordance of 83%. This discrepancy may depend not only to *in silico* prediction algorithm but also to differences in additional evidences contributing to the final scores, since the compared variants were not belonging to the same familiar and clinical contexts.

What we learned from our work is that ADPKD genetics is complicated by the relative high frequency of non-truncating variants, the high allelic heterogeneity with many private variants, the presence of multiple variants in the same patient. In this scenario, segregation analysis may be fundamental at least to exclude the pathogenic role of a new variant. Unfortunately, in the present study, contribution of segregation studies for unclassified variants was limited because collection of samples from the families was done more often when a truncating variant was present.

In our opinion, to harmonize and improve assessment of variant pathogenicity for ADPKD it would be important to define a consensus based on standards and possibly on validated criteria. This goal could be supported by improving data-basing and sharing of data and possibly by constituting a multidisciplinary panel of experts, as already experienced for other human diseases (i.e. InSiGHT’s Variant Interpretation Committee)^17^. Databasing is becoming a very important resource for the clinic and for the research; in particular it may be fundamental in the classification and stratification of clinical phenotypes and to not disperse the amount of data that are continuously produced by clinical laboratories.

## Patients and Methods

### Patients

The analysis of PKD genes was performed in a cohort of 440 unrelated Italian patients with ADPKD and 203 family relatives, for a total 643 subjects. Among the 440 patients, 320 with a familial positive history, 120 patients with no reported familiarity. Patients were collected in a period of seven years; all the examined subjects gave their written informed consent for genetic testing, data treatment and storage, approved by the Institutional Quality Assessment committee. All the methods were performed in accordance with the guidelines and the experimental protocols approved by the San Raffaele Hospital Istitutional Quality Assessment committee (IQNet IT-2337). In Table 9, phenotypic details on our study patients are reported (age, gender, sCr, eGFR and CDK stages, 1 to 5, at the time of testing). Also available data on extra-renal manifestations associated with ADPKD (hypertension and liver cysts) are reported in Table 9. The patients were referred to the genomic facility from the recruiting groups according to three main modalities and diagnostic questions: i) patients with a family history of ADPKD and positive diagnostic criteria (Research Track); ii) patients with a positive family history at risk for ADPKD but not responding to clinical diagnostic criteria (Diagnostic Track); iii) patients without a family history but a suggestive renal cystic phenotype (Differential Diagnosis Track).

The clinical criteria in the presence of a family history were derived with minor modifications from previous works^18^,^19^. In brief, in the presence of a positive family history, patients were considered affected if the ultrasound reported 3 cysts or more unilaterally or bilaterally in the age class 15–39 years; more than two cyst per each kidney for subjects in the age class 40–59 years; four or more cysts in each kidney was required for individuals older than 60 yr^18^. For patients analyzed by MRI in the presence of positive family history more than 10 cysts as total number in both kidneys was needed to confirm the diagnosis in patients older than 15 years^19^.

### Genetic analysis

Genetic analysis was performed on genomic DNA extracted from peripheral blood on a DNA automatic extractor (Maxwell, Promega, Madison, WI) with the DNA purification kit (Promega), following the manufacturer’s instructions.

#### Sanger direct sequencing

For all the patients, *PKD1* and *PKD2* (MIM-601313 and 613095) whole coding regions and exon junctions were analysed by directly sequencing PCR products with a Sanger protocol, on both strands. To amplify the functional *PKD1*, avoiding amplification from pseudo-genes, we used a first round of Long Range PCR using primers and conditions previously described^7^ and specific for the functional *PKD1*. The 8 Long Range PCR products were then amplified in 43 nested reactions to obtain PCR amplified products corresponding to exons 1–33 and exon-intron junctions. Exons 34 to 46 of PKD1 as well as the 15 exons of PKD2 were amplified from genomic DNA in a single PCR round because these regions are not duplicated in pseudo-genes. PCR conditions and primers are listed in Supplementary Table S9; new PKD1-specific primers for nested PCR have been designed taking into account a multiple alignment (Lasergene) between PKD1 and the 6 pseudogenes.

PCR, nested PCR and sequencing reactions were set up on a liquid handling system (Biomeck FX, Beckman-Coulter) according to protocols developed in the laboratory. PCR and sequencing reactions were purified with Ampure and CleanSeq (Agencourt, Beckman-Coulter) on the FX platform. Dye terminator reaction sequences were loaded on a 3730 AB (Applied Biosystems Inc., Foster City, CA) automatic sequencer. Called sequences were aligned to the reference ENSG00000008710 and ENSG00000118762 with the Sequencer v.5.0 (Gene Codes Corporation, Ann Arbor, MI). Gene variants were named according to the standard nomenclature (Human Genome Variation Society, www.hgvs.org). The Autosomal Dominant Polycystic Kidney Disease Data Base, public genomic browsers (Ensembl, NCBI, 1000Genomes) and data published in the literature were searched to look for already described variants and for variant classification. All of the sequence changes identified have been confirmed on a second DNA extraction from the same blood sample.

#### Multiple Ligation Probe Assay

In patients with no variants detected with Sanger sequencing, a MLPA analysis has been performed with the MRC-Holland SALSA MLPA P351 PKD1 and P352 PKD1-PKD2 probe-mix, following the manufacturer’s instructions.

Identification of deletion breakpoints was performed by specific amplification of deletion borders and direct sequencing.

#### RNA analysis

We used Paxgene™ Blood RNA System (PreAnalytiX, Hombrechtikon, CH), which consists of an evacuated Paxgene™ RNA tube (PAX tube) for blood collection and a processing kit (PAXgene Blood RNA Kit, Qiagen) for isolation of total RNA from whole blood.

1 μg RNA was reverse transcribed into cDNA with random hexamer using MuLV reverse transcriptase (Roche) and amplified by PCR using *PKD1* exonic primers: forward 5′-GCGTCTGAGCCGTGAAG -3′ and reverse 5′-GCCCAGGCAGCCGCAGT -3′ located in exons 9 and 11 respectively, and yielding an amplicon of 585 bp. Direct sequencing of the amplicon was carried out as described above.

### Classification of variants

Variants identified in the present study were classified as previously described^9^: i) large rearrangements, nonsense, frameshift deletions, insertions or indels, variants affecting canonical splice-sites, in-frame changes of ≥5 amino acids were classified as definite pathogenic variants; ii) in-frame changes of <5 amino acids, missense and atypical splicing previously reported in patients, segregating with the disease were classified as highly likely pathogenic variants; iii) novel missense variants with a combined score ≥14 and intronic variants at positions +3 and −3 from the exon were classified as likely pathogenic variants; iv) novel missense variants with a combined score <14 were classified as variants of uncertain significance; v) novel synonymous variants and deep intronic variants were classified as likely non pathogenic; vi) known common or rare variants previously classified as likely neutral were classified as benign variants.

To give a meaning to missense variations, we applied the criteria adopted by Audrezet and co-authors^9^, using different prediction software: the Grantham matrix scoring system Align Grantham Variation Grantham Deviation (A-GVGD)^20^, PolyPhen2^21^,^22^, Sorting Intolerant from Tolerant (SIFT)^23^,^24^, and Mutation Taster^25^. All of them base their prediction on phylogenetic and structural information. They use as input file both the protein itself (PolyPhen2 and SIFT) searching automatically for orthologs and homologs, or a list of orthologous proteins (*Homo sapiens*, *Rattus norvegicus*, *Gallus gallus*, *Mus musculus*, *Takifugu rubripes*, *Danio rerio*) previously chosen (SIFT and AGVGD). To strengthen the results, also the conservation scores PhyloP^26^ and phastCons^27^,^28^ have been calculated. Moreover, the segregation of the variant in affected members of the family raises the pathogenicity.

For known missense variants, a comparison of the scores obtained and their previous classification from the ADPKD database, (http://pkdb.mayo.edu)^4^, was performed.

### Statistical analyses

We examined ESRD by Chi-square testing in subjects with different classes of pathogenic variants in *PKD1* and *PKD2*. We compared the age at ESRD by Kaplan-Meier analysis with Log-rank testing in subjects with pathogenic variants in *PKD1* and *PKD2*.

### Databasing

As a database for the collected *PKD1* and *PKD2* gene variants we selected the internationally linked LOVD version 3 platform. LOVD is structured in agreement with all existing guidelines of the HGVS and HVP^29^ and allows collection, annotation and classification of all variants detected, as well as storing key clinical data, described based on the Human Phenotype Ontology (HPO) phenotypic descriptors^30^. Use of the HPO descriptors may warrant a higher homogeneity especially when data are shared among a number of clinics. The databases can be accessed using the urls http://www.LOVD.nl/PKD1 and http://www.LOVD.nl/PKD2 and through LOVD’s webservice (api). The structure, documentation, and operability of the DB are available on-line.

The complete list of contents is reported in Table 10. Contents refer to variant and patient data and are mostly public with the exception of some individual annotations as well as identification codes. Clinical departments contributed individual and clinical data.

## Additional Information

**How to cite this article**: Carrera, P. *et al*. Deciphering Variability of PKD1 and PKD2 in an Italian Cohort of 643 Patients with Autosomal Dominant Polycystic Kidney Disease (ADPKD). *Sci. Rep*. **6**, 30850; doi: 10.1038/srep30850 (2016).

## Supplementary Material

Supplementary Information

Supplementary Table S1

Supplementary Table S2

Supplementary Table S3

Supplementary Table S4

Supplementary Table S5

Supplementary Table S6

Supplementary Table S7

Supplementary Table S8

Supplementary Table S9

## Figures and Tables

**Table 1:** Total number of PKD1 and PKD2 variants identified, grouped in classes.

Cases	PKD1		PKD2	
	Familial	Sporadic	Familial	Sporadic
Large rearrangements	2	1	0	1
Canonic splicing	17	7	8	2
Frameshift	73	19	10	0
Nonsense	60	17	15	3
In frame indels ≥5	4	0	0	0
In frame indels <5	13	8	1	0
Missense	165	51	12	6
Intronic	87	24	10	3
Synonym	54	23	3	2
*Total*	475	150	59	17
Total F + S	625		76	

**Table 2:** PKD1 and PKD2 novel variants identified, grouped in classes.

Cases	PKD1		PKD2	
	Familial	Sporadic	Familial	Sporadic
Large rearrangements	0	1	0	0
Canonic splice sites	8	5	3	1
Frameshift	46	12	5	0
Nonsense	26	9	5	2
In frame indels ≥5	3	0	0	0
In frame indels <5	6	2	0	0
Missense	104	36	6	5
Intronic	67	19	10	3
Synoyim	46	19	2	1
*Total*	306	103	31	12
Total F + S	409		43	

**Table 3:** Distribution of definite and probable pathogenic variants in 352 probands.

Type of variant		*PKD1*		*PKD2*	
Definite pathogenic					
Large rearrangements		3	1%	1	2%
Frameshift		92	30%	10	19.5%
Nonsense		77	26%	18	35%
Canonic splicing		24	8%	10	19.5%
In-frame ≥5 aminoacids		4	1%	0	
Probable pathogenic (HLP + LP)					
Missense		75	25%	9	18%
In-frame <5 aminoacids		20	7%	1	2%
Atypical splicing		6	2%	2	4%
**Pathogenic variants spectra, comparison with previous large studies**					
	Present study n = 352*	CRISP n = 180*	GENKYST n = 442*	TGESP n = 188*	**HALT-PKD CRISP n** = **1034**^**§**^
PKD1					
truncating	196 (55.7%)	103 (57.2%)	286 (64.7%)	72 (38.3%)	592 (57.3%)
nontruncating	81 (23%)	41 (22.8%)	73 (16.5%)	51 (27.1%)	223 (21.6%)
IF indel	24 (6.8%)	9 (5%)	29 (6.6%)	8 (4.3%)	54 (5.2%)
PKD2	51 (14.5%)	27 (15%)	54 (12.2%)	57 (30.3%)	165 (15.9%)
**Classification of PKD1 and PKD2 variants and detection rates**					
	***PKD1***		***PKD2***		***PKD1***** + *****PKD2***
	**DP**	**HLP + LP**	**DP**	**HLP + LP**	
Familial (n = 320)	154 (48%)	73 (23%)	33 (10%)	6 (2%)	266 (83%)
Sporadic (n = 120)	46 (38%)	28 (24%)	6 (5%)	6 (5%)	86 (72%)
*Total* DP	200		39		239 (54%)
Total HLP + LP		101		12	113 (26%)
	*Total PKD1* 301 (85.5%)		*Total PKD2* 51 (14.5%)		352 (80%)

*°*All in-frame indels grouped together.

*Total probands/families with pathogenic variants.

^§^Total patients with pathogenic variants truncating: frameshift, nonsense, canonic splice site, large rearrangements nontruncating: missense, atypical splicing.

DP: definite pathogenic; HLP: highly likely pathogenic; LP: likely pathogenic.

**Table 4:** Variants with a discrepant classification.

*PKD1*												
DNA change (cDNA)	protein change	mutation type	exon/IVS	Gs	AGVGDs	PP2s	SIFTs	MTs	total score	concurrent variants/classification	prediction in this study	PKDB classification
c.224C>T	p.Ser75Phe	missense	2	155	C25	B	D	D	12	no	uncertain significance	highly likely pathogenic
c.827C>T	p.Thr276Ile	missense	5	89	C0	B	T	T	2	p.Glu2771Lys/HLP p.Leu726Phe/US p.Asp1332Asn/US	uncertain significance	likely pathogenic
c.1606G>A	p.Gly536Ser	missense	7	50	C0	D	T	D	8	no	uncertain significance	likely pathogenic
c.5830G>A	p.Gly1944Arg	missense	15	125	C0	D	D	D	14	p.Ala3053Thr/LP	likely pathogenic	indeterminate
c.6062T>C	p.Leu2021Pro	missense	15	98	C0	D	D	D	14	no	likely pathogenic	indeterminate
c.6151C>T	p.Arg2051Cys	missense	15	180	C15	B	T	T	3	c.11537 + 2T>A/DP p.Arg3277Cys/Hypomorphic	uncertain significance	likely pathogenic
c.7271C>T	p.Thr2424Met	missense	18	81	C65	D	D	D	20	no	likely pathogenic	indeterminate
c.9047A>G	p.Gln3016Arg	missense	25	43	C35	D	T	D	11	no	uncertain significance	highly likely pathogenic
c.9361G>A	p.Glu3121Lys	missense	26	56	C55	D	T	D	13	p.Arg4154Cys/HLP	uncertain significance	likely pathogenic
c.9412G>A	p.Val3138Met	missense	27	21	C15	D	D	D	13	no	uncertain significance	likely pathogenic
c.10678G>A	p.Gly3560Arg	missense	36	125	C0	D	T	D	14	p.Phe3257Leufs*57/DP	likely pathogenic	likely neutral
c.10951G>A	p.Gly3651Ser	missense	37	56	C55	D	T	D	13	p.Arg2765His/US	uncertain significance	highly likely pathogenic
c.11258G>A	p.Arg3753Gln	missense	39	43	C0	D	T	D	8	p.Pro1429Ser/US	uncertain significance	highly likely pathogenic
c.12826C>T	p.Arg4276Trp	missense	46	101	C0	D	D	D	14	p.Leu56Argfs*15/DP	likely pathogenic	indeterminate

DP: definitely pathogenetic; HLP: highly likely pathogenic; LP: likely pathogenic; US: uncertain significance.

**Table 5:** Pathogenic variants found in at least 3 unrelated patients.

cDNA	Protein	Exon/IVS	Number of unrelated patients	Prediction	Described/Novel
*PKD1*					
c.856_862delTCTGGCC	p.Gly287fs*1	5	4	definitely pathogenic	Described
c.2180T>C	p.Leu727Pro	11	4	likely pathogenic	Described
c.5014_5015delAG	p.Arg1672Glyfs*97	15	9	definitely pathogenic	Described
c.3607C>T	p.Gln1203*	15	3	definitely pathogenic	Novel
c.8311G>A	p.Glu2771Lys	23	5	highly likely pathogenic	Described
c.8935_8937delTTC	p.Phe2979del	24	3	highly likely pathogenic	Described
c.11134delC	p.Arg3712Glyfs*14	38	3	definitely pathogenic	Described
c.11354G>C	p.Trp3785Ser	40	3	likely pathogenic	Novel
c.6916-9G>A		IVS15	3	highly likely pathogenic	Described
c.8017-2_-1delAG		IVS21	3	definitely pathogenic	Described
*PKD2*					
c.637C>T	p.Arg213*	2	6	definitely pathogenic	Novel
c.916C>T	p.Arg306*	4	3	definitely pathogenic	Described
c.2159dupA	p.Asn720Lysfs*5	11	3	definitely pathogenic	Described
c.1094 + 1G>A	p.?	IVS4	3	definitely pathogenic	Described

**Table 6:** Patients with multiple novel variants.

	PKD1	PKD2	PKD1 + PKD2
N. of patients with 1 variant	216	70	
N. of patients with 2 variants	118	3	
N. of patients with 3 variants	39	0	
N. of patients with 4 variants	14	0	
N. of patients with variants in both genes			45

**Table 7:** Cases with more than 1 variant in *PKD1, PKD2* and classified as pathogenic.

N.	CDNA	Protein	Trans	Score	Prediction for novel variants	Novel/PKDB classification
	***PKD1***					
1	c.8689_8690insCCAACTCCG	p.Val2897delinsAlaAsnSer	y		highly likely pathogenic	novel
	c.12112insG	p.Val4038Glyfs*118			definitely pathogenic	novel
2	c.7859_7863delCGAG	p.Asn2620Ilefs*39			definitely pathogenic	novel
	c.7863 + 1_ + 4delGTGA				definitely pathogenic	novel
3	c.6050C>A	P.Ser2017*				definitely pathogenic
	c.12442G>A	p.Glu4148Lys		17	likely pathogenic	novel
4	c.9694A>T	p.Lys3232*			definitely pathogenic	novel
	c.10651C>T	p.Pro3551Ser		20	likely pathogenic	novel
5	c.11537 + 2T>A				definitely pathogenic	novel
	c.9829C>T	p.Arg3277Cys				likely hypomorphic
6	c.6005_6043del39	p.Val2002_Gln2014del			definitely pathogenic	novel
	c.4057G>A	p.Gly1353Ser		19	likely pathogenic	novel
7	c.2098-2_2109del14				definitely pathogenic	novel
	c.9739C>T	p.Arg3247Cys		20	likely pathogenic	novel
8	c.7119C>G	p.Cys2373Trp		20	likely pathogenic	novel
	c.8405C>T	p.Pro2802Leu				likely pathogenic
9	c.11017-10C>A					highly likely pathogenic
	c.8369C>T	p.Pro2790Leu		16	likely pathogenic	novel
	c.12454A>G	p.Lys4152Glu		17	likely pathogenic	novel
	***PKD1 *****+***** PKD2***					
10	*PKD1* c.8293C>T	p.Arg2765Cys				likely hypomorphic
	*PKD1* c.10043G>A	p.Arg3348Gln				likely pathogenic
	*PKD2* c.1094 + 1G>A					definitely pathogenic
11	*PKD1* c.6583_6589del7	p.Cys2195Glyfs*15				definitely pathogenic
	*PKD2* c.2392C>T	p.Arg798Cys				likely pathogenic

**Table 8:** Correlation between genotype and ESRD.

	PKD1			PKD2
	T*	NT°	IF indel°	All°
ESRD n = 44	28 (64%)	2 (4.5%)	3 (6.8%)	3 (6.8%)
no ESRD n = 132	62 (47%)	20 (15.2%)	3 (2.3%)	21 (16%)

*Chi-square, p-value = 0.055.

°Not significant.

**Table 9:** Characteristics of the study cohort.

Total number of study patients	643
Total number of study families/probands	440
Mean age, yr (SD)	42 + /−20
Gender	
Men	227 (52%)
Women	213 (48%)
CDK stage n = 234	
1	80 (34%)
2	61 (26%)
3	28 (12%)
4	9 (4%)
5	56 (24%)
Extra-renal manifestations	
Hypertension n = 129	75 (58%)
Liver cysts n = 133	81 (61%)
Families/probands with no variants	25 (6%)
Families/probands with VUS	63 (14%)
Families/probands with pathogenic variants	352 (80%)
Families/probands with PKD2 pathogenic variants	51 (12%)
men	26 (51%)
women	25 (49%)
truncating	39 (11%)
not-truncating	11 (3.1%)
IF indel	1 (0.3%)
Families/probands with PKD1 pathogenic variants	301(68%)
men	152 (50%)
women	149 (50%)
truncating	196 (55.7%)
not-truncating	81 (23%)
IF indel	24 (6.8%)

*eGFR calculated from serum creatinine measurements, milliliters/minute per 1.73 m^2^.

**Table 10:** Database Contents.

*PUBLIC DATA*		
GENERAL INFO	PREDICTIONS	KEY CLINICAL DATA
Gene name	Grantham	End Stage Renal Disease
Reference sequence	A-GDGV	(ESRD)
Links	Polyphen2	Type of Renal Replace Therapy
	SIFT	Renal cysts
VARIANT INFO	Mutation Taster	Extra-renal cysts
Exon	Effect	Cerebral berry aneurism
DNA change (cDNA)		Hypertension
DNA change (genomic)	PATIENT INFO	Macrohematuria
Published as	Gender	Nephrolithiasis
RNA change	Geographic Origin	Creatinine level (mg/dl)
Protein	Ethnic Origin	Ellipsoid US volume
DB ID	Consanguinity	Ellipsoid MRI volume
Allele transmission	Inheritance	Multi-slice MRI volume
Variant remarks	Disease	
Pub Med reference	Disease status	
Frequency	Age at diagnosis	
Re-site	Age at ESRD	**NON-PUBLIC DATA**
Genetic origin	Age of death	Individual ID
Segregation		Family ID
dbSNP rs		Clinic ID
Template		Individual remarks
Technique		
Tissue		
