# Supplementary material for: Deciphering Variability of PKD1 and PKD2 in an Italian Cohort of 643 Patients with Autosomal Dominant Polycystic Kidney Disease (ADPKD)
Source: Sci Rep. 2016 Aug 8;6:30850. doi: 10.1038/srep30850 (PMC4976333; doi:10.1038/srep30850)
Supplement: Supplementary Information [file srep30850-s1.docx]

Supplementary

DECIPHERING VARIABILITY OF PKD1 AND PKD2 IN AN ITALIAN COHORT OF 643 PATIENTS WITH AUTOSOMAL DOMINANT POLYCYSTIC KIDNEY DISEASE (ADPKD)

Paola Carrera* ^1,2^, Silvia Calzavara ^2^, Riccardo Magistroni ^3, 6^, Johan T. den Dunnen ^10^, Francesca Rigo^1^, Stefania Stenirri ^1^, Francesca Testa ^6^, Piergiorgio Messa ^5^, Roberta Cerutti ^5^, Francesco Scolari ^7^, Claudia Izzi ^7^, Alberto Edefonti ^9^, Susanna Negrisolo^11^, Elisa Benetti^12^, Maria Teresa Sciarrone Alibrandi ^4^, Paolo Manunta ^4^, Alessandra Boletta ^3^, Maurizio Ferrari ^1,2,8^

**^1^** IRCCS San Raffaele Scientific Institute, Division of Genetics and Cell Biology, Unit of Genomics for Human Disease Diagnosis, Milan, Italy; **^2^** Laboratory of Clinical Molecular Biology, Ospedale San Raffaele, Milan, Italy; **^3^** IRCCS San Raffaele Scientific Institute, Division of Genetics and Cell biology, Molecular Basis of Polycystic Kidney Disease Unit, Milan, Italy; ^4^Vita-Salute San Raffaele University, chair of Nephrology, IRCCS Ran Raffaele Scientific Institute, Genomics of Renal Disease and Hypertension Unit, Milan, Italy; **^5^** Dept. of Nephrology, Urology and Transplant, IRCCS Cà Granda Policlinico, Milan, Italy; **^6^** Division of Nephrology and Dialysis A.O. U. Policlinico, University of Modena and Reggio Emilia, Modena, Italy; **^7^** Center for Prenatal Diagnosis and Nephrology, A.O. Spedali Civili, Brescia, Italy, **^8^**Vita-Salute San Raffaele University, chair of Clinical Pathology, Milan, Italy; ^9^ Dept. of Paediatric Nephrology and Dialysis, IRCCS Cà Granda Policlinico, Milan, Italy; ^10^Depts. Clinical Genetics and Human Genetics, Leiden University Medical Centre, Netherlands; ^11^Laboratory of Immunopathology and Molecular Biology of the Kidney, Dept. SDB, Padova, Italy; ^12^Pediatric Nephrology, Dialysis and Transplant Unit; Department of Women’s and Children’s Health, Padova, Italy.

*Correspondence to: Paola Carrera, IRCCS San Raffaele Scientific Institute, via Olgettina 60, 20132 Milano, Italy. E-mail: carrera.paola@hsr.it


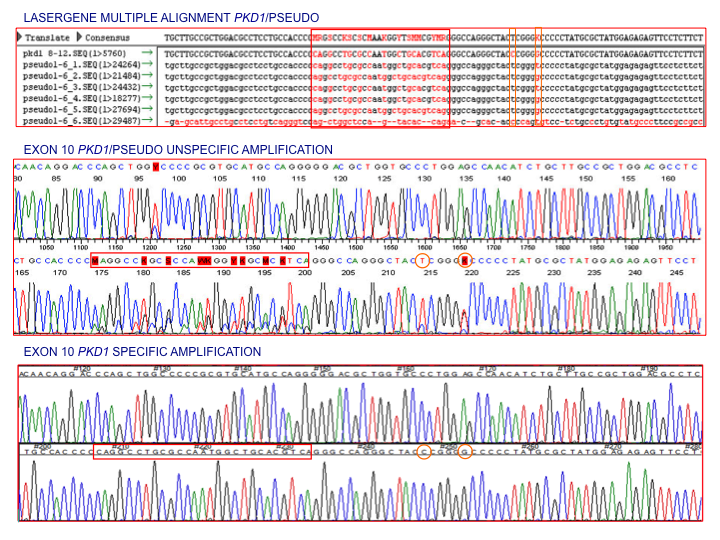


Supplementary Figure S1.

Specificity of amplification of *PKD1* duplicated region. To avoid amplification of the *PKD1* pseudogenes, the design of primers was verified on a multiple alignment (Lasergene) between *PKD1* and the 6 pseudogenes. In the upper panel the multiple alignment is shown for a portion of *PKD1* exon 10. Red boxes highlight regions of diversity between aligned sequences. In the middle panel, sequencing results on a not-specific amplification is shown, with the regions boxed showing the presence of multiple peaks; in the lower panel, sequencing results using PCR specific primers with the regions boxed showing the presence of single peaks, identifying univocally the *PKD1* gen.
